# Strategies to Overcome Erroneous Outcomes in Reverse Transcription-Polymerase Chain Reaction (RT-PCR) Testing: Insights From the COVID-19 Pandemic

**DOI:** 10.7759/cureus.72954

**Published:** 2024-11-03

**Authors:** SM Shafiul Alam Sajal, Dewan Zubaer Islam, Shahad Saif Khandker, Elizabeth Solórzano-Ortiz, Manal Fardoun, Md Firoz Ahmed, Mohd. Raeed Jamiruddin, Nafisa Azmuda, Miral Mehta, Santosh Kumar, Mainul Haque, Nihad Adnan

**Affiliations:** 1 Department of Biochemistry and Molecular Biology, Jahangirnagar University, Dhaka, BGD; 2 Department of Microbiology, Jahangirnagar University, Dhaka, BGD; 3 Department of Microbiology, Gonoshasthaya Samaj Vittik Medical College, Dhaka, BGD; 4 Department of Chemical, Biological, Biomedical and Biophysical Research, Mariano Gálvez University, Guatemala City, GTM; 5 Department of Pharmacology and Toxicology, Faculty of Medicine, American University of Beirut, Beirut, LBN; 6 Department of Pharmacy, Bangladesh Rural Advancement Committee (BRAC) University, Dhaka, BGD; 7 Department of Pedodontics and Preventive Dentistry, Karnavati School of Dentistry, Karnavati University, Gandhinagar, IND; 8 Department of Periodontology and Implantology, Karnavati School of Dentistry, Karnavati University, Gandhinagar, IND; 9 Department of Pharmacology and Therapeutics, National Defence University of Malaysia, Kuala Lumpur, MYS

**Keywords:** clinical, contamination, coronavirus, covid-19, detection, false-negative, false-positive, molecular diagnostics, rt-pcr, sars-cov-2

## Abstract

The reverse transcription-polymerase chain reaction (RT-PCR) test to detect SARS-CoV-2, the virus causing COVID-19, has been regarded as the diagnostic gold standard. However, the excessive sensitivity of RT-PCR may cause false-positive outcomes from contamination. Again, its technical complexity increases the chances of false-negatives due to pre-analytical and analytical errors. This narrative review explores the elements contributing to inaccurate results during the COVID-19 pandemic and offers strategies to minimize these errors. False-positive results may occur due to specimen contamination, non-specific primer binding, residual viral RNA, and false-negatives, which may arise from improper sampling, timing, labeling, storage, low viral loads, mutations, and faulty test kits. Proposed mitigation strategies to enhance the accuracy of RT-PCR testing include comprehensive staff training in specimen collection, optimizing the timing of tests, analyzing multiple gene targets, incorporating clinical findings, workflow automation, and implementing stringent contamination control measures. Identifying and rectifying sources of error in RT-PCR diagnosis through quality control and standardized protocols is imperative for ensuring quality patient care and effective epidemic control.

## Introduction and background

**Figure 1:**
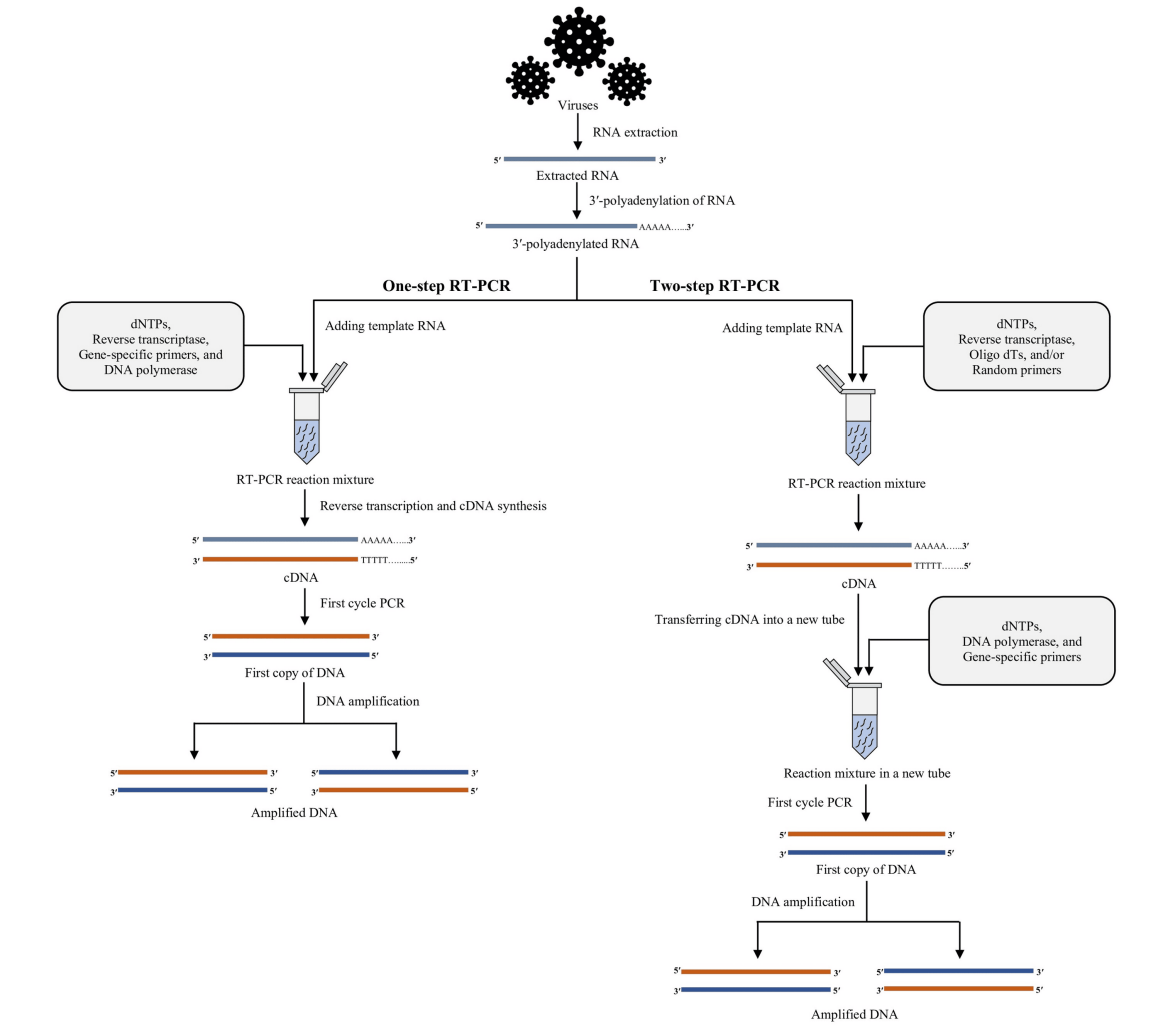
Illustration showing the steps in RT-PCR. RNA: ribonucleic acid; DNA: deoxyribonucleic acid; cDNA: complementary deoxyribonucleic acid; dNTP: deoxynucleotide triphosphate; PCR: polymerase chain reaction; RT-PCR: reverse transcription-polymerase chain reaction; oligo dTs: oligonucleotide deoxy-thymidines Image credit: Shahad Saif Khandker

Molecular diagnosis is the medical testing discipline that employs a set of molecular methods. Molecular diagnostics aims to improve detection, diagnosis, sub-classification, prognosis, and therapeutic response monitoring by identifying certain genetic traits [1]. The development of nucleic acid probe technology was made feasible by our growing knowledge of the chemistry of nucleic acid hybridization [2]. Now that nucleic acid amplification technology exists, it is possible to identify and characterize microorganisms without first cultivating them. For many species that are hard to detect, molecular approaches are now the most reliable means of detection. The nucleic acid amplification through the in vitro method has been simplified and expedited with the advent of the polymerase chain reaction (PCR) and other established amplification techniques [1,3]. In 1993, for the first time, Higuchi developed a straightforward, quantitative method for any amplified DNA sequence; this method came to be known as real-time polymerase chain reaction (PCR) [4]. Molecular biology has revolutionized diagnostics by improving the care of infected patients, particularly against infectious diseases [5]. Since 1980, after the discovery of PCR, much research work has been done, making it possible to use molecular tests in many areas of routine clinical microbiology [4]. Molecular biology uses these analytical tools in many techniques and various ways [5]. Real-time reverse transcription-polymerase chain reaction (RT-PCR) is an increasingly crucial diagnostic technique in diagnostic laboratories [4]. The term "RT-PCR" refers to a process in which complementary DNA (cDNA) is produced from RNA through reverse transcription (RT). Subsequently, a particular cDNA is amplified using a polymerase chain reaction (PCR) (Figure 1) [4]. This method of detection and quantification of mRNA is currently the most efficient and accurate. Virus detection and microbial cell viability testing are two of the most common applications of RT-PCR.

Through this approach, the PCR products are detected, confirmed, and quantified in real-time utilizing fluorescent-labeled probes. PCR-based systems that can detect genetic variants that may cause a specific disease from clinical samples without the need to grow them in a lab have been helpful for quickly finding microorganisms that cannot be grown in a lab or are hard to grow [6]. Also, subsequent analysis of amplified microbial DNA makes it possible to track the pathogen and learn more about it. Different methods have been used to find subspecies-level differences, essential in the prognosis and treatment of some diseases. Other necessary steps include figuring out how much virus is in the body/determining pathogen load and finding the genes or mutations in genes conferring important phenotypes, i.e., drug resistance [6]. The application of automation and easy-to-use software has made it usable for more and more laboratories with personnel without broader expertise.

Coronaviral disease 2019 (COVID-19), initiated through the virus named severe acute respiratory syndrome coronavirus 2 (SARS-CoV-2), has substantially impacted diagnostic microbiology. Researchers have applied many detection techniques to meet growing demands and combat the devastating pandemic. These are determined by virological testing using RT-PCR, in which swabs (i.e., nasopharynx and oropharynx), saliva, feces, and a chest radiograph are utilized and monitoring the levels of inflammatory mediators, such as cytokines, in real-time [7]. RT-PCR is the most used technique for detecting messenger RNA (mRNA) and quantification techniques that can determine the existence of coronavirus genes in a biological specimen [8]. The RT-PCR test is the gold standard for COVID-19 disease diagnosis in clinical and research settings [9]. Because of the closed system in which amplification and detection occur, real-time PCR techniques reduce diagnosis time and contamination hazards [4]. However, false-negative and false-positive results were reported in RT-PCR molecular diagnosis. Many diagnostic laboratories in low- and middle-income countries (LMICs) did not have the necessary infrastructure or trained technical personnel to develop and interpret RT-PCR tests; however, the massive need for tests as a result of the increase in COVID-19 infections at the beginning of the pandemic imposed the need to accelerate the implementation of these tests, with all the challenges that this implied, among these: differences in sensitivity of commercial tests, the lack of knowledge on the limit where the cycle threshold (CT) values indicate an infectious viral load, and the absence of correlation of the patient's clinical information in the CT values interpretation [10]. In this review article, the authors comprehensively discussed the reasons behind the false-positive and false-negative RT-PCR results, particularly during the COVID-19 pandemic, along with the strategies and essential precautionary steps to mitigate generating false results in RT-PCR-mediated diagnosis. 

Problem statement of this study

There are devastating consequences to false-positive and false-negative molecular test results, especially during a pandemic like COVID-19. When an incorrect test result misleads a doctor, the patient risks not obtaining the care they need or waiting too long for treatment. Patients can experience psychological damage from needless and intrusive follow-up testing [11]. This issue is not just theoretical; in March 2020, the Centers for Disease Control and Prevention in the United States revoked testing kits because they saw an elevated rate of false-positive reports due to reagent contamination [12]. On the other hand, if an infectious disease like COVID-19 is not correctly diagnosed, it may spread more rapidly. False-positive results before surgeries, unnecessary cancellation of treatment or postponement, and urgent admissions pose a higher chance of infection if the patient follows the incorrect course in hospital settings [9]. False-positive results have also created severe problems in the management and treatment of some harmful diseases, like breast cancer. Vohra et al. conducted a study in Pakistan and found that 4.9% of asymptomatic patients had COVID-19 before surgery [13]. The false-negative result was also inauspicious for these kinds of patients. Wei et al. assessed the clinical features of COVID-19-affected breast cancer patients as well as the risks associated with anti-cancer treatment. When compared to other breast cancer patients, individuals who started chemotherapy within seven days of the initiation of COVID-19 symptoms had a significant correlation with COVID-19 severity, along with severe neutropenia [13]. False-negative test findings might do more considerable harm than false-positive ones. Persons infected with a virus like SARS-CoV-2 but getting negative results will do their normal daily activities and spread the disease to their family members and others in their surroundings [14]. RT-PCR is widely recognized as the gold standard for diagnosing COVID-19, providing rapid and accurate virus detection. However, low and middle-income countries (LMICs) have encountered significant challenges in implementing RT-PCR testing. The healthcare crisis exacerbated by emerging variants of SARS-CoV-2, coupled with insufficient infrastructure and untrained personnel, has led to the occurrence of false-positive and false-negative results. These inaccuracies have had severe repercussions for patient care and disease control, resulting in unnecessary treatments and further spread of the disease.

Proper steps are necessary to mitigate the false-positive and false-negative test results and enable the physicians and other clinical staff to be more confident regarding adequate patient treatment. Moreover, in cases like COVID-19, reducing erroneous results may decrease the frequent spreading of coronaviruses like infectious diseases.

Objectives of this study

**Figure 2:**
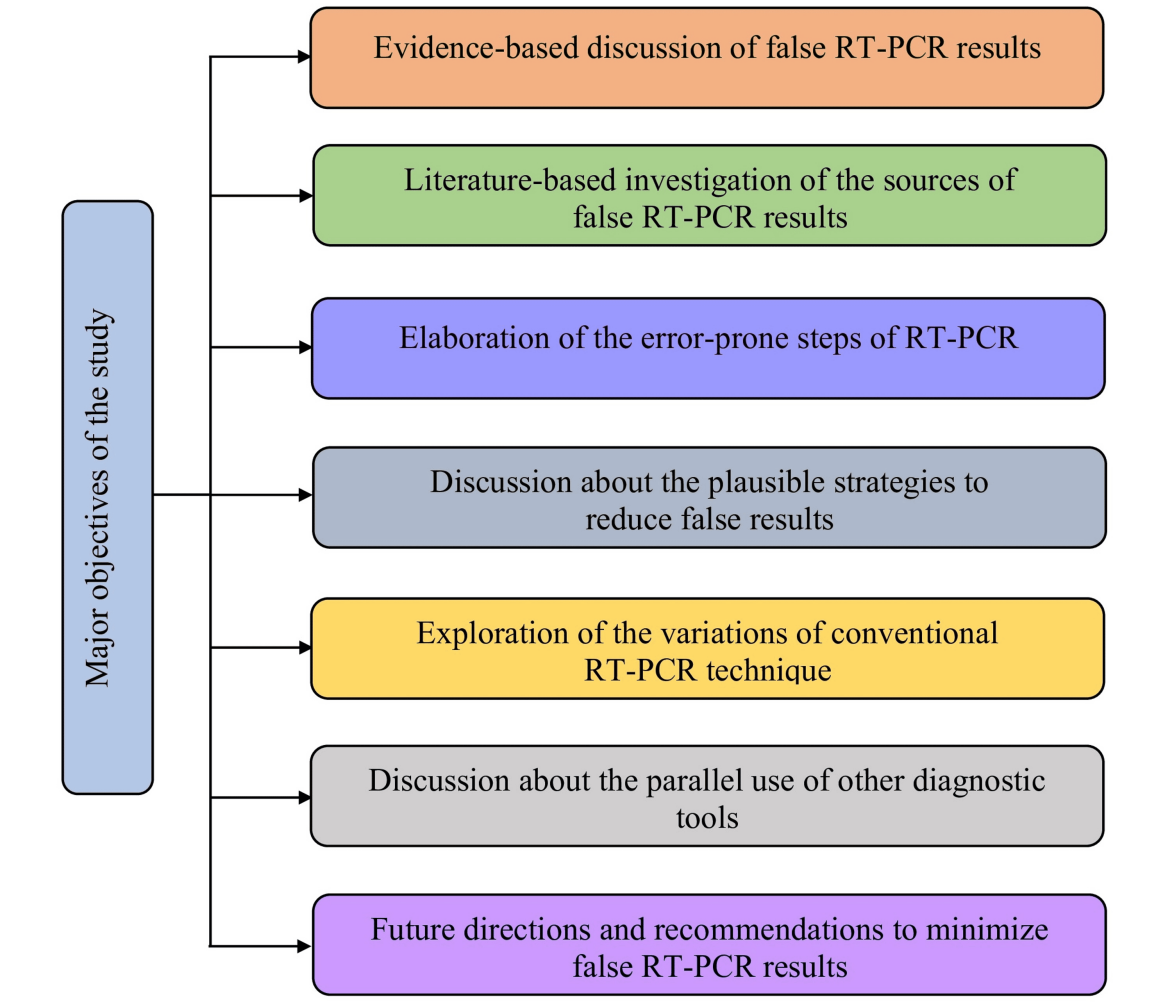
Major objectives of the study. mRNA: messenger ribonucleic acid; cDNA: complementary deoxyribonucleic acid; PCR: polymerase chain reaction; RT-PCR: reverse transcription-polymerase chain reaction Image credit: Shahad Saif Khandker

This narrative review aims to point out the potential sources and reasons that may lead to the generation of false-positive or false-negative results in RT-PCR testing and to comprehensively discuss the strategies to minimize erroneous RT-PCR results based on the information obtained from the previously published articles and relevant sources (Figure 2).

## Review

Materials and methods

**Figure 3:**
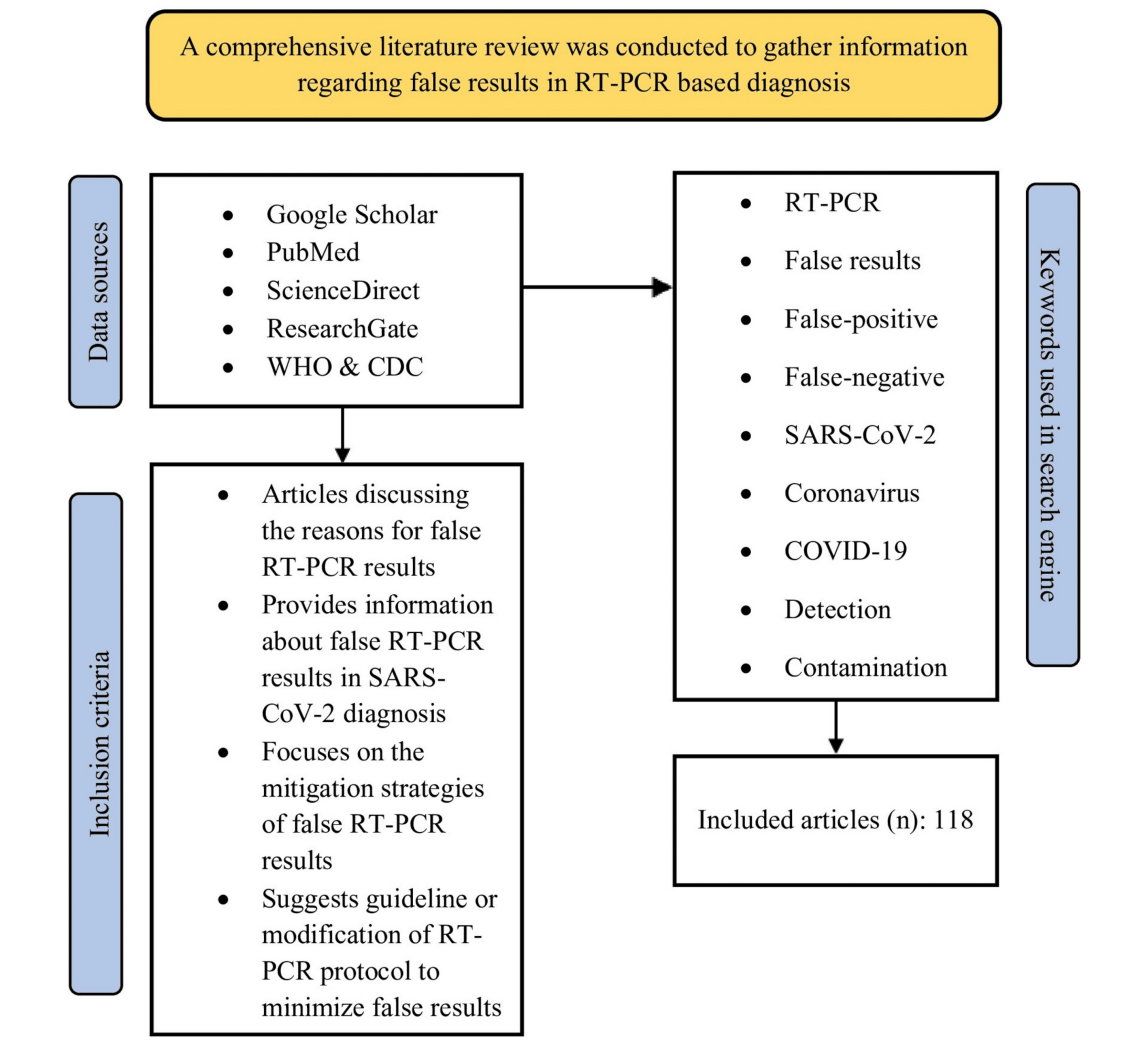
Schematic diagram illustrating the methodology of the study. WHO: World Health Organization; CDC: Centers for Disease Control and Prevention; RT-PCR: reverse transcription-polymerase chain reaction; n: number of included articles Image credit: Dewan Zubaer Islam

Since the sole aim of this review study was to discuss the reasons for false results in RT-PCR testing, particularly for the diagnosis of SARS-CoV-2 during the COVID-19 pandemic, relevant information was searched out and extracted from articles available in three major online databases, e.g., Google Scholar, ScienceDirect, and PubMed, using specific keywords, e.g., "RT-PCR," AND "false-positive," AND "false-negative," AND "false-results," AND "SARS-CoV-2", AND "coronavirus," AND "COVID-19", AND "detection," AND "contamination," etc. The search keywords were applied in different combinations and adjusted by the Boolean operators (AND, OR). Relevant data were also collected from articles available in ResearchGate, as well as various recommendations, guidelines, and investigation reports published by the World Health Organization (WHO) and the Centers for Disease Control and Prevention (CDC) (Figure 3). The authors carefully selected the articles and extracted information to be included in the review. No restrictions regarding publication year limit, specific study design of the articles, or article type, e.g., original research, review articles, systematic review articles, meta-analyses, book chapters, case reports, correspondence, news, short communications, mini-reviews, editorials, etc. were applied by the authors during searching articles. Only articles written in English were decided to be included in this review.

Review of literature

Factors Contributing to False-Positive Results

**Figure 4:**
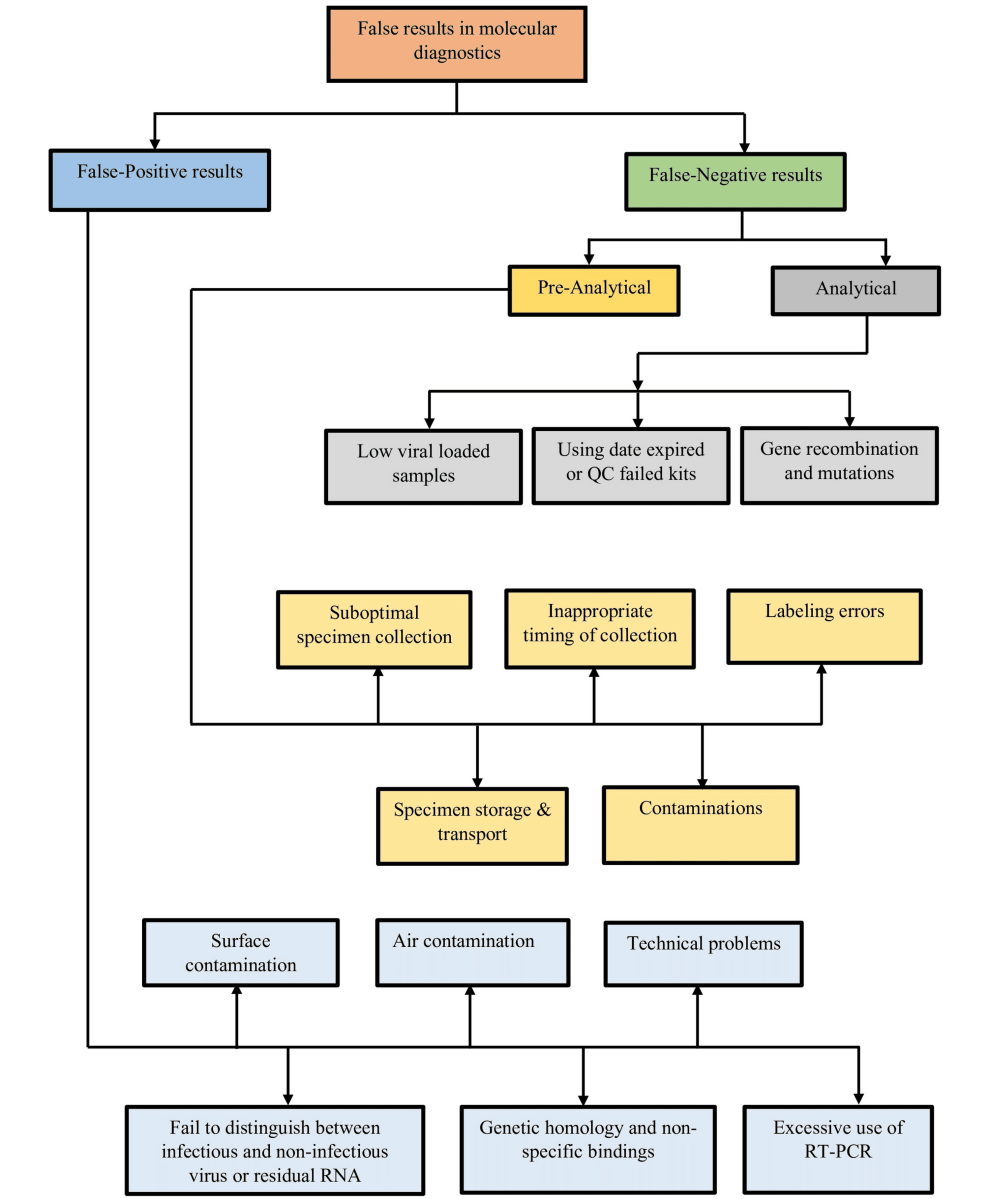
Block diagram of false-positive and false-negative results in molecular diagnostic. QC: quality control; RNA: ribonucleic acid; RT-PCR: reverse transcription-polymerase chain reaction Image credit: Dewan Zubaer Islam

When an actual negative result is wrongly diagnosed as positive, this condition is known as a false-positive [15]. The first false-positive findings were published in 1988 by Lo et al., where it was reported that PCR primers designed to detect the hepatitis B virus (HBV) had been contaminated with plasmid DNA, which contained a full-length HBV insert [16]. A false-positive test incorrectly identifies a specific infection as engaged, but the condition is not current [14]. Therefore, positive test outcomes do not always confirm the presence of a disease or virus in the body. RT-PCR is the procedure that collects results throughout the PCR process as it amplifies the DNA or processes RNA through reverse transcription. There is an initial recognition of target amplification. There is also a point where the fluorescence intensity becomes more significant than the background fluorescence; that time is known as the threshold cycle (Ct) [17]. Viral load is inverse to the Ct value, characterized by higher viral load in clinical specimens correlating to lower Ct values shown in RT-PCR [18]. Suppose the viral load is higher for a more extended duration in patients severely affected during regular PCR testing; after a period, false-positive might occasionally be identified for these cases because of having an inactivated virus in the body [18]. Some common factors contribute to false-positive results in the molecular laboratory (Figure 4). These include contamination from earlier amplified products, the existence of exogenous target DNA in the water, chemical reagents, and kits, as well as in the sterile blood culture material, a poorly designed primer, poor amplicon conditions, and contamination caused by the people working in the laboratory [4,19].

Surface contamination: This may happen in the sample collection room and laboratory working areas, especially during the diagnosis of COVID-19. The most probable zones of surface contamination are the sample collection booth, sample processing or extraction area, and template addition area. The hospital area where the infected patients are treated is also the most common zone of surface contamination [20,21]. Viral and bacterial contamination is widespread and often occurs if improperly handled. A COVID-19-infected person can quickly spread the virus around the area where he resides and the sample collection room. Mouchtour et al. collected environmental samples from a ferryboat, a nursing home, and a hospital ward where COVID-19 patients were isolated with proper safety during an ongoing inquiry into a COVID-19 outbreak. They detected RNA of SARS-CoV-2 on the cover of the air drain duct, a screen for the hospital, ship, and cabin air release that opened onto the deck. Infected persons have displaced respiratory droplets/nuclei on the air ducts and the ship's air ventilation system evacuation screen on the open deck [22]. Many suspected COVID-19 cases had to come to the sample collection area for sampling. They might spread the surface of the sample collection area. If a genuinely negative person provides an example of an environmentally contaminated area, their result may be falsely positive [22]. If surface contamination occurred in the laboratory where the diagnosis was performed, it could have been responsible for false-positive results [14].

Air contamination in the sample collection area and the laboratory: Although a symptomatic patient has been reported to manifest broad surface contamination with SARS-CoV-2, little is known regarding SARS-CoV-2 transmission through the air [21,23]. The obliviousness of the sample collectors, the laboratory personnel, and the infected patients can contaminate the surface area and the air of the sample collection room and the laboratory. One study examined the air in patients' cabins and bathrooms oriented toward the identical air exhaust duct. These individuals were either sick or asymptomatic. They also discovered that SARS-CoV-2 RNA was present on the filter surface and inside the air conditioning unit. They detected viral RNA in one of the 12 air samples [16]. These observations indicated that the airflow might have moved droplets or nuclei emerging from the respiratory systems of infected patients [24]. So, if the positive sample or positive control is exposed in the laboratory room, it could contaminate the air and give false-positive results. Inhalation of vaccine aerosols by vaccinators may also generate false-positive results if their nasopharyngeal or oropharyngeal samples are diagnosed by RT-PCR [25].

Technical errors: False-positive findings can be attributed to technical issues such as contamination during the sample processing period; for instance, if the unknown sample accidentally contacts a contaminated hand or surface or contamination of PCR amplicons and reagents [9]. Contaminations of commercial primers/probe sets can also contribute to false-positive results [19]. Operator errors, e.g., improper placement of specimens in a testing plate, incorrect plate formatting, and cross-contamination from the specimens that have a high viral load with low viral specimens, especially in adjacent wells of test plates, are the common reasons behind false-positive RT-PCR results, as reported by Layfield et al. [26]. Cross-contamination increases when collecting or working with a large number of samples. This is especially true in two-step RT-PCR, where RNA extraction and polymerization are performed in separate tubes [27]. During molecular testing, it is possible to get false-positive results due to cross-reactions with different viruses or other genetic material, as well as cross-contamination of negative samples with positive ones and positive ones with negative ones [9].

Error in distinguishing infectious and non-infectious viruses or residual RNA and high sensitivity: Lan et al. found positive RT-PCR results in patients who had already recovered from COVID-19; however, the test was incapable of distinguishing between live viruses and non-infectious viruses or residual RNA, and as a consequence, the result was misled towards being false-positive [7,28]. RT-PCR tests can detect inactive or active viruses and give positive results. However, the clinical specimens are at such a low concentration that they cannot cause infection or any harm to the body. Amongst them were endogenous and exogenous factors, such as blood or nasal spray ions or compounds that replicated the test cassette's pH, which could influence the test results. It has the potential to provide erroneous positive findings [29].

Genetic homology and non-specific bindings: Genetic homology among microorganisms and non-specific bindings of nucleic acids during molecular testing make it difficult to identify true positives. Because the targeted areas of the genes in both SARS-CoV-2 and SARS-CoV have similarities, Van Kasteren et al. discovered that some tests could identify both virus strains. In some cases, the similarity between SARS-CoV-2 and other infections, respiratory tract organisms, or colon organisms might lead to false-positive findings [27]. Fluorescence, primer dimers, probes, short oligonucleotide primers, or fluorescent dyes that engage nonspecifically with double-stranded DNA (dsDNA) or even single-stranded DNA (ssDNA) might be to blame for producing false-positive findings in RT-PCR. In addition, diverse techniques utilize various genes and probes, some of which may not be equal. Therefore, the limits of detection (LoD) for those numerous assays can vary by a factor of one hundred [30]. The widespread usage of rapid diagnostic tests (RDTs) required personnel to have more relevant skills and adequate knowledge about RT-PCR laboratory processes and reasons for false-positive results [14]. False-positives may also be generated for cross-reaction. Serological tests generally diagnose dengue in dengue-endemic areas where cross-reaction of dengue samples in COVID-19 serological tests or vice versa causes misinterpretation. Research has found 14% cross-reactivity within dengue virus and SARS-CoV-2 antibodies during serological testing [31].

Excessive use of RT-PCR: To meet the current demand, increased RT-PCR use by untrained persons and inadequate RT-PCR laboratory processes maximize the likelihood of producing false-positive results [14]. Other reasons for false-positives are inadequate laboratory experience, probes, types of fluorescence in the kits, and a positive person performing the detection procedure [14]. Even if a person does not have an active SARS-CoV-2 infection during testing, COVID-19 nasal swab test results may indeed be influenced and appear indecisive if SARS-CoV-2 nucleic acid contamination enters the nasal pathway through inhalation or touching the face with contaminated hands. These individuals might not have any symptoms and may not have had any contact with a COVID-19-positive person [32]. The chance of false-positive results increases when too much target DNA is handled when real-time PCR standard curves are set up or when positive control templates are used [33]. Mislabeling during collection or processing, contamination while sampling and processing the specimen, and low-quality reactions in PCR may generate false-positive results [34].

Factors Contributing to False-Negative Results

When a test wrongly indicates that the pathogenic condition does not exist, it is known as a false-negative [35]. People infected with negative test results do not know whether they are sick since they are unaware of their infection status. Based on the results of their tests, they can build a false feeling of security. These individuals threaten further transmission of the illness, which might increase the disease's prevalence [14]. The rate of false-negative for SARS-CoV-2 in respiratory specimens has varied between 1% and 30%, depending on the study [36]. In a study examining the features of SARS-CoV-2 conversion in 70 COVID-19 patients, 21.4% of the patients experienced a "turn positive" of nucleic acid detection by RT‐PCR test after two consecutive negative findings [37]. A systematic review and meta-analysis report by Pecoraro et al. revealed that in RT-PCR tests, up to 58% of COVID-19 patients have a risk of getting false-negative results [38].

**Figure 5:**
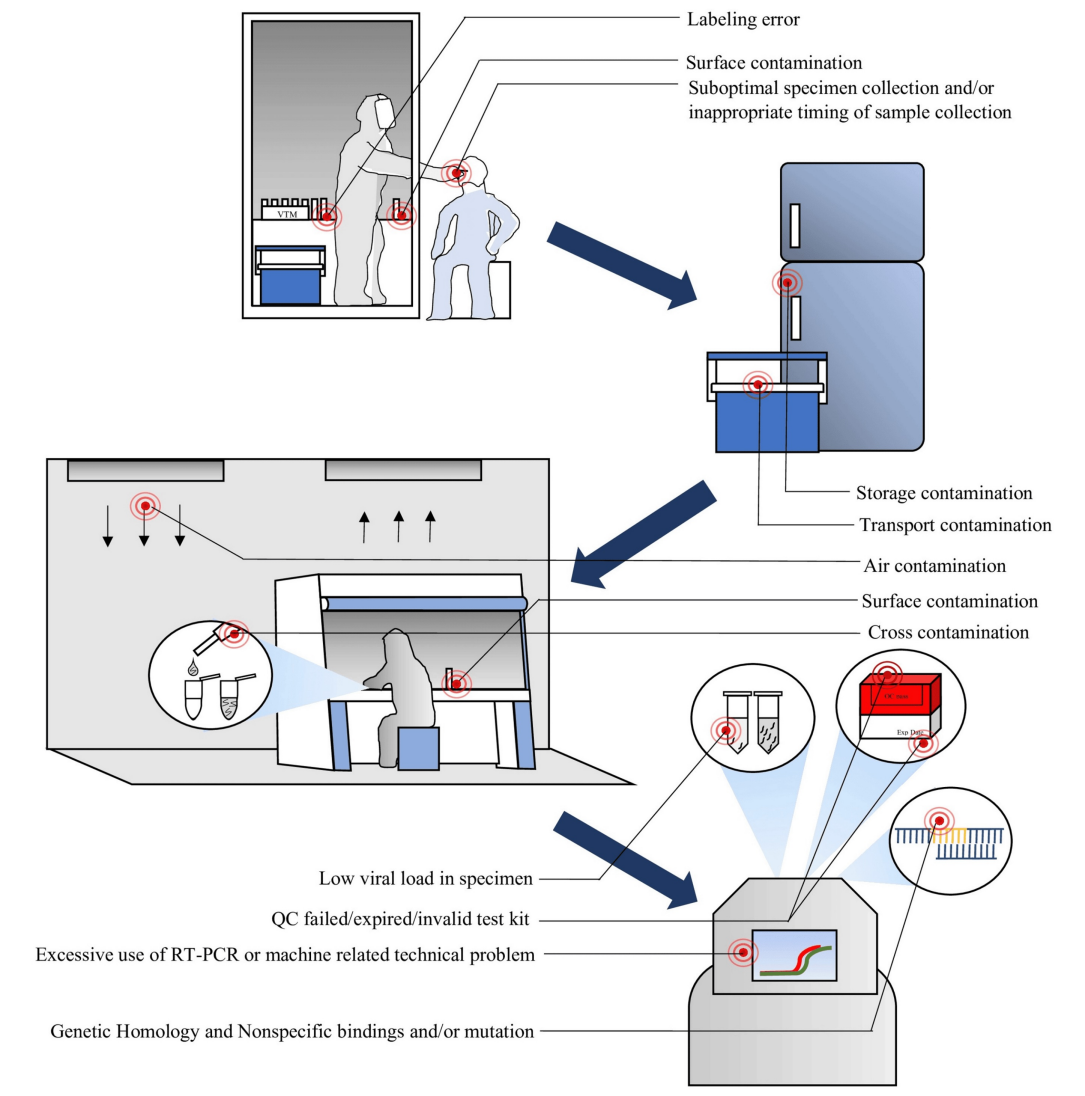
Possible reasons for false-positive and false-negative RT-PCR results. QC: quality control; RT-PCR: reverse transcription-polymerase chain reaction Image credit: Shahad Saif Khandker

The possibility of false-negative results in molecular diagnostics depends on many factors, mainly sampling and technical reasons [39]. Researchers have hypothesized two primary causes of detection failures: preanalytical and analytical errors [40]. Some common factors that are responsible for false adverse outcomes in the molecular laboratory are the presence of PCR inhibitors in the reactions; improper extraction, handling, and storage techniques; accidental loss of the template nucleic acid target; nucleic acid template digestion by endogenous DNases and RNases; poorly designed primers; having low sensitivity and specificity of nucleic acid; and common amplicon conditions (Figure 5) [4]. Also, false-negative results may occur because of faulty transportation of specimens, insensitivity of the testing platform caused by inherent shortcomings, misidentification, too-early or too-late sampling, viral load kinetics in different anatomic sites, genetic diversity, and low-level inadvertent contamination during sample collection and processing [41-45]. For example, Lomas et al. reported that false-negative PCR results could be caused by the unintentional introduction of glove powder into the tube while changing the gloves to reduce false-positive results caused by contamination. However, it is widely acknowledged that using powdered gloves constitutes poor practice in handling specimens intended for subsequent processing in molecular biology [45].

Pre-Analytical Factors

Suboptimal specimen collection: In molecular diagnostics, when nasopharyngeal swabs are frequently requested for respiratory viruses to look for SARS-CoV-2, findings regarding false-negative results might be attributed to improper specimen collection. Because it includes inserting the swab into the posterior nasopharynx, the collection of a specimen of excellent quality is a task that calls for prior training and experience. After inserting the swab to a depth of around 7 centimeters, the user will rotate and remove it [46]. Inadequate nasopharyngeal sampling, especially in the presence of nasal obstructions and sampling by untrained personnel, may cause false-negative results [47]. Zimmer et al. commented that inadequate sampling, e.g., taking a throat swab instead of a nasopharyngeal swab, may lead to false-negative RT-PCR results [41]. A team measured the amount of human deoxyribonucleic acid (DNA) extracted from nasopharyngeal swabs to evaluate the possibility that inadequate sample collection was the root of false-negative test findings. Human DNA might act as a reliable molecule-based identifier of the overall quality of the specimens gathered [48]. The study observed significantly low levels of human DNA in nasopharyngeal swab samples suspected to be false-negative, compared to a group of samples taken in order where the introductory period of the test was similar [48]. According to this observation, even in the lowest percentile of specimens, the human ribonuclease P-RNA level was still well enhanced before the critical threshold of 40. Still, the threshold may not be adequate for improperly collected samples to provide accurate results [48,49].

Inappropriate timing of sample collection: Another reason people could obtain a false-negative test result even though they might genuinely be positive for a virus is that they might collect their samples at the wrong time of the infection cycle. This can happen if they test too early in their illness or too late in their infection. Several prior studies have provided narratives of individuals who were subjected to testing. AI and colleagues accomplished a retrospective study using 1,014 patients affected by SARS-CoV-2. At the time of the initial submission, RT-PCR results were negative for 413 (41%) of these individuals [50]. Xie et al. did the same thing, looking at 167 infected patients. At the initial presentation, RT-PCR results were negative for five (3%) patients [51]. When evaluating swabs that were obtained zero to six days after the beginning of symptoms, Fang et al. discovered that RT-PCR could identify only 36 out of 51 (71%) of the patients infected by SARS-CoV-2 [52]. Liu et al. and Zhao et al. concluded that the percentage of infected patients with positive test results decreased with time after their symptoms began [51,53]. Song and Pan et al. stated that viral load could be smaller than the limit of quantitation (LoQ) during the primary and late stages of infection [54,55]. Strong immunity against SARS-CoV-2 infections, notably among young patients, may also result in low viral loads in samples that may eventually cause false-negative RT-PCR results [54].

Labeling errors: Errors in labeling can lead to incorrect medication or therapy withholding in patients. Misidentifying a patient or the patient's material can be an error that may occur during the labeling process for laboratory specimens [56]. It is also possible that the place from which the material was acquired is incorrectly identified, and mistakes of this type might lead to considerable patient uneasiness and even injury. Only a few studies have been done to document the error rate in the anatomical pathology laboratory [56]. A research group reviewed an experiment with wrongly labeled specimens for 18 months in a laboratory and calculated the error percentage per case. The research found 75 labeling mistakes, representing 0.25% of the total instances. Of these 75 errors, 55 (73%) included the patient's name, 18 (24%) related to the location, and the majority of the mislabeling, 52 (69%), took place in the laboratory [57]. In the specific case of COVID-19, labeling errors were more frequent during peak cases due to the large number of samples being tested simultaneously and the collapse of health systems operating beyond their capacities. An inappropriate specimen that is not labeled can create confusion at the time of testing and may result in false-negatives during molecular testing.

Specimen storage and transportation: In the LMICs, the tests were centralized in the main cities at the pandemic's beginning. Hence, the samples had to be transported long distances, and maintaining the cold chain during transportation represented a challenge [10]. A study took 30 COVID-19-positive samples and divided them into two groups for storage: one at 4°C and another at room temperature. They also classified the positives into three categories based on the CT values: low (CT value <25, number of cases n: 12), medium or moderate (CT value 25-32, n: 11), and high (CT value 32-38, n: 7) [58]. They discovered one high CT sample at ambient temperature was negative after 24 hours, and one sample at 4°C was negative after four days. On day 12, they discovered seven positive specimens from the ambient temperature group, and two positive specimens from the 4°C group were false-negative [58]. However, after three days, the CT values of the specimens from the 4°C group were almost unchanged [58]. The respiratory specimens should be maintained between 2- 8°C until 72 hours following collection. If a delay in the testing or transportation is anticipated, the specimens must be kept in a cold environment, maintaining a temperature of -70 °C or lower [59]. A false-negative result may be obtained from the sample if the temperature is not correctly controlled. Inadequate sample transportation and storage conditions, such as a faulty cold chain and extended time spent in transit, can produce false-negative findings [40,60]. Essential medium (MEM), phosphate-buffered saline (PBS), saline, and viral transport medium (VTM) should indeed be utilized during the duration of transport or storage [61]. Swab samples should be collected in an appropriate tube that is either vacant or consists of either viral transport medium (VTM), Amies transport medium, or sterile saline, as per the recommendations of the CDC and WHO for SARS-CoV-2 tests. These recommendations state that the swab samples must be put in a proper transport tube [59]. Otherwise, the rate of false-negatives may increase.

Contaminations: Because whole blood freezing and the use of improper additives might cause interference with tests, the sample may contain components that could cause problems, such as the release of cellular particles [62]. During the processing of samples, a variety of operational issues may occur. Pipetting mistakes can occur during the manual preparation of samples or the aliquoting of the specimens or reagents. The laboratory may experience several points during testing, including cross-contamination and sample mismatching [63]. Because of these issues, the accuracy of the molecular tests can be called into question. Even trace quantities of foreign DNA in a sample might lead to inaccurate test results if the model is contaminated [62].

Analytical Factors

Low viral-loaded samples: Low viral loads in collected samples are among the major causes of false adverse RT-PCR outcomes. Swab samples collected from the nasopharynx may generate false-negative RT-PCR results due to inadequate viral particle quantities in these areas [64]. The RT-PCR method is relatively sensitive, but it has some limitations. A clinical investigation observed that a subset of individuals with fever and indicative manifestations of viral pneumonia, specifically lower lobe lung lesions, initially tested negative for the virus in throat swab nucleic acid tests employing RT-PCR. The viral presence was not confirmed until five to six days following the onset of viral pneumonia. These cases were identifiable through chest computed tomography (CT) scans despite the virus remaining undetected through the tests above [65]. Noteworthy is the documentation of approximately 60% of SARS-CoV-2 infections being asymptomatic, with anticipation that merely 30 to 60% of COVID-19 patients could yield positive findings, subsequently established through chest CT and supplementary diagnostic methodologies [66,67]. A recent cohort study conducted at a residential university in Bangladesh indicated a reduced ability of RT-PCR to detect asymptomatic COVID-19 infections [68].

Expired or QC-failed kits or reagents and invalid assays: An experimental group discovered that the specificity of an OraQuick ADVANCE® kit (OraSure Technologies, Inc., PA, USA) with one month to expiration decreased significantly, implying that an expired or QC-failed kit was associated with a false-negative result [69]. Inadequate material, low quality or volume, and invalid assays may lead to erroneous results [40]. Most medical and diagnostic products have an expiration date to ensure their reliability and effectiveness. Using an expired kit can compromise the integrity of the reagents or components, leading to unreliable results. If a reagent fails quality control (QC), it may not meet the required specifications or standards for reliable and accurate results. The reliability of results obtained using that kit would be questionable or compromised. Quality control is essential in ensuring the accuracy and precision of laboratory tests. When a reagent fails QC, it suggests that the kit may not perform as intended. Using a reagent that cannot maintain QC may lead to inaccurate or unreliable results, potentially impacting the validity of experiments or diagnostic tests. Depending on the design of the test, it might give either a false-positive or negative diagnosis [59].

Mutations of genes and diagnostic accuracy: The occurrence of false-negative RT-PCR results can be attributed to the lack of a "standardized universal primer(s)" (SUP). Different regions and nations use different primers and probes for RT-PCR testing, leading to discrepancies caused by virus mutations at the primer annealing site [70]. In one approach, the E gene is used as the primary screening assay after a corroboratory testing method using an RNA-dependent RNA polymerase (RdRp) assay. Kits, e.g., GeneFinder (OSANG Healthcare, South Korea), which has been widely used since 2020 and approved by the FDA, already used the N gene as a confirmatory assay [40]. Concerning the CDC test, three sets of N gene-specific primers and probes are intended for widespread identifications of SARS-CoV-2. About the CDC test, three N gene primer/probe sets are intended for widespread identifications of SARS-like coronaviruses and to determine SARS-CoV-2. In addition, another set of primers and probes is designed to detect the human RNase P gene (RP) in control samples. These are the components that make up the test [71].

Another significant problem that has come to light is the possibility of active gene recombination and mutation. Mutation at the target site has occurred in different SARS-CoV-2 variants, which is responsible for generating false-negative results due to the failure of primer-probe binding to the targeted site during detection. Research suggests alterations in the spike proteins of the beta variant may facilitate evading the immunological response mechanism [72]. Artesy et al. documented that Roche has linked a cytosine-touracil (C-to-U) mutation at position 26340 of the SARS-CoV-2 genome to the malfunction of the cobas system. The cobas system utilizes a dual-target quantitative reverse transcription polymerase chain reaction (qRT-PCR) assay designed to detect both the ORF1ab region and the E gene of the SARS-CoV-2. This mutation leads to false-negative results for the E gene qRT-PCR while yielding positive results for the ORF1ab region [73]. Hasan et al. have disclosed a newly identified point mutation within the N gene at C29200A of the SARS-CoV-2 genome, which has been found to impact the detection of the virus using the Cepheid Xpert Xpress SARS-CoV-2 (Xpert) assay (Cepheid, CA, USA), an FDA-approved qRT-PCR-based test for COVID-19 [74].

Additionally, Zieglar et al. have reported a single nucleotide polymorphism (SNP) at C29200T in the N2 region of SARS-CoV-2, which has been observed to affect detection utilizing the N2 primer/probe set of the Cepheid Xpert Xpress SARS-CoV-2 assay [75]. Vanaerschot et al. have indicated that a single polymorphism at G29140U in the forward N gene primer binding site could also potentially compromise the sensitivity of RT-PCR, thereby implicating an adverse impact on viral detection [76]. The error-prone RNA-dependent RNA polymerases found in coronavirus are likely to blame for any mutations. Shen et al. reported notable findings relating to the diversity of viral strains within infected individuals, identifying a median of 4 inter-viral variations. This observation indicates the rapid evolution of SARS-CoV-2. In a separate study, Yi discovered up to five distinct SARS-CoV-2 haplotypes, suggesting active genetic recombination [77,78]. Such viral evolution likely contributes to variations in immune response, virulence, pathogenicity, and transmissibility.

Moreover, mutation rate fluctuations may compromise RT-PCR detection accuracy [79,80]. Other analytical causes of false-negative results are tests performed outside the diagnostic opportunity, disconnected primers and probes, non-specific PCR annealing, misinterpretation of the results, and mechanical errors [40]. Many risk factors are responsible for false-positive and false-negative results. Some common factors may cause both, and some are specific to only one false result (Table 1).

**Table 1:** Major risk factors and sources of false-positive and false-negative results.

Risk factors	Possibility of occurring (false-positive results)	Possibility of occurring (false-negative results)
Contaminations	Yes	Yes
Technical problems	Yes	Yes
High sensitivity	Yes	No
Genetic homology and non-specific bindings	Yes	No
Genetic mutations	No	Yes
Suboptimal specimen collection	No	Yes
Labelling errors	Yes	Yes
Storage and transport	No	Yes
The low viral-loaded sample	No	Yes
Invalid assay	Yes	Yes
Excessive test	Yes	Yes
Residual RNA	Yes	No
Misinterpretation of the results	Yes	Yes

Mitigation Strategies for False-Positive and False-Negative Results

**Figure 6:**
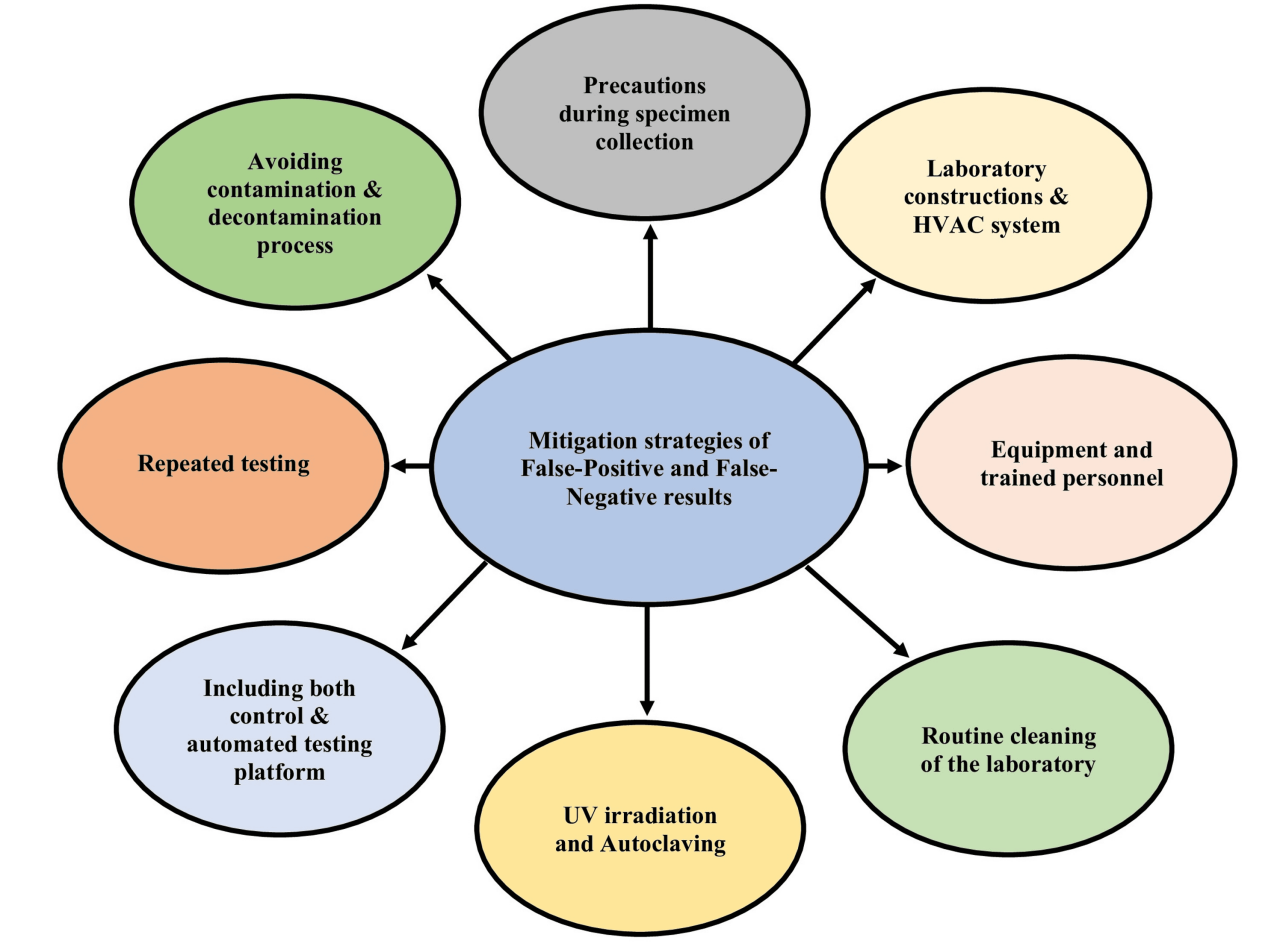
Mitigation strategies for false-positive and false-negative RT-PCR results. HVAC: heating, ventilation, and air conditioning; UV: ultraviolet; RT-PCR: reverse transcription-polymerase chain reaction Image credit: Dewan Zubaer Islam

False-positive and false-negative diagnoses have detrimental implications for patients, doctors, and others [34,81]. Accurate diagnostic assays for SARS-CoV-2 have raised a significant obstacle to COVID-19 diagnosis. It is the primary barrier, particularly in a case that requires urgent treatment [14]. Accurate laboratory testing is necessary for diagnosing and treating infectious illnesses, especially given these conditions' rapid emergence and spread [35]. We cannot stop the spread of COVID-19 if we fail to interpret it correctly in the molecular laboratory. We must learn how it occurs and the mitigation strategies to reduce false-positive and false-negative results of these molecular diagnoses (Figure 6).

Collecting adequate specimens: A trained worker is required during sample collection to reduce collection errors. For COVID-19, the insertion of a swab is around 7 cm in depth, followed by rotation and revocation of the swab. Material should be collected from both the nasopharyngeal and oropharyngeal cavities using swabs featuring a synthetic tip, such as nylon or Dacron, according to the guidelines provided by the Centers for Disease Control and Prevention (CDC) in the United States [82]. Specimens from the lower respiratory tract can be collected when the collection device has a shaft made of aluminum or plastic [82]. The swab should be inserted into the nose parallel to the palate, held in place for a few seconds, and placed in the oropharyngeal cavity to allow for secretion and absorption. Following collection, the swab should be put into a sterile tube containing 2-3 mL of viral transport media [40]. During oropharyngeal (e.g., throat) specimen collection, it was noted that swabbing the posterior pharynx during this time with the tongue should be avoided and that rapid insertion of the swab into another different viral transport medium should be performed. Failing to follow the methods provided might be a substantial source of diagnostic mistakes [83]. Utilizing samples from the lower respiratory tract, such as bronchoalveolar lavage (BAL) or bronchial aspirates, may reduce the incidence of false-negative outcomes owing to the abundance of viral loads in these areas. However, this approach necessitates invasive methodologies such as patient intubation, thereby restricting its widespread applicability [64]. A recent study by Chen et al. has elucidated the efficacy of a well-designed microneedle patch comprising two distinct cross-linked clusters in conjunction with SARS-CoV-2 spike-specific antibodies, demonstrating enhanced tissue penetration and efficient virus capture. This microneedle patch containing modified heads of regular oropharyngeal swabs reduces the risks of false-negative results caused by inefficient sampling and swabs containing low viral loads [84]. Specimens collected from multiple anatomic sites, e.g., collecting stool and blood samples alongside respiratory samples, can reduce the chances of generating false-negative results [44,85].

Laryngotracheal aspiration test (LTA) can be applied instead of nasopharyngeal swabs (NPS), as LTA showed more feasibility and safety and reduced false-negative results during RT-PCR testing. When tested on suspected SARS-CoV-2 patients, the LTA method outperformed serial nasopharyngeal swabs and bronchoalveolar lavage (BLA) and had a higher negative predictive value than nasopharyngeal swabs [86]. Zhang et al. have emphasized the imperative nature of cellular materials, specifically infected host cells, in detecting SARS-CoV-2 and mitigating false adverse outcomes. They have proposed the utilization of Ct cutoff values for the RPP30 gene to anticipate false-negative RT-PCR results for SARS-CoV-2 RNA, ensuring heightened sensitivity (95.03%-95.26%) and specificity (83.72%-98.55%) [87].

Proper timing of specimen collection: In a study by Kucirka et al., it was noted that the false-negative rate reaches its lowest point three days after the onset of symptoms or approximately eight days after exposure. Consequently, clinicians should wait one to three days after the manifestation of symptoms to minimize the probability of obtaining false-negative RT-PCR results [88]. Additionally, the appropriate collection of specimens within seven days of the onset of clinical symptoms such as ageusia and anosmia, as well as the meticulous administration of swabs by trained technicians, have been found to significantly decrease the occurrence of false-negative RT-PCR results [89].

Optimization of primers/probes: False RT-PCR results may occur due to using unoptimized primers, a lack of "standardized universal primer(s)," and extensive variations in the mutations of primer-probe annealing sites in the SARS-CoV-2 DNA. Park et al. highlighted the propensity for unoptimized primer sets to yield false-positive results in their study. They proposed a systematic three-step approach for designing and optimizing specific primer sets to address this. This approach involves the selection of primer sets for target genes, particularly the RdRP, N, E, and S genes within the SARS-CoV-2 genome, in silico validation of primer and amplicon sequences, and the optimization of PCR conditions, such as primer concentrations, annealing temperatures to facilitate specific hybridization, and the mitigation of primer dimers [90]. Using a combination of primers can be a good option in these cases. For instance, Tsutae et al. reported that using a combination of primers (JPN-N2 and CDC-N2) reduced false-negative results most effectively among the four sets of primers they used, namely JPN-N1 ('Japan's N1 primer), JPN-N2, CDC-N1, and CDC-N2 [70].

Performing additional tests alongside RT-PCR: A computed tomography (CT) scan can be performed as an additional test with routine, conventional RT-PCR to reduce false-negative results due to its enhanced sensitivity and rapid turnaround time [91]. Xu et al. suggested considering CT scan results, routine hematological tests, and examining clinical manifestations to predict false-negative RT-PCR results. They added the significant association of "ground glass opacity" in CT imaging with a lower risk of generating false-negative results. In contrast, consolidation, basophils, and eosinophils were linked to an increased risk of false-negative results, indicating the significance of routine hematologic examination [92].

Several hematological parameters, including aspartate aminotransferase and lactate dehydrogenase levels, vary significantly among COVID-19-positive and negative patients. Routine analysis of these parameters can dramatically reduce false RT-PCR results [93]. Serological assays, particularly those based on enzyme-linked immunosorbent assay (ELISA), can minimize the limitations of RT-PCR by facilitating the detection of asymptomatic SARS-CoV-2 infections and viral RNA in later stages of infection diagnosis [31]. The integration of immunoassay-based microfluidic kits and other biosensor devices presents significant potential for use alongside RT-PCR owing to their heightened sensitivity, specificity, and point-of-care testing (POCT) capabilities, thereby reducing the likelihood of yielding false results [5]. For instance, a flow-through dot-blot immunoassay-based microfluidic kit coupled with gold nanoparticles developed by Sil et al. exhibited notable sensitivity, specificity, and decreased detection time in comparison to in-house ELISA or chemiluminescence immunoassay methods [94].

Variation in RT-PCR platform and procedure: Sometimes conventional PCR platforms and the testing procedure often generate false results. However, RT-nested PCR, a modification of traditional RT-PCR, showed higher sensitivity with reduced numbers of false-negative results compared to the real-time PCR technique [95]. Digital PCR, renowned for its accuracy and high sensitivity, can detect infections from oropharyngeal or nasal swab samples due to its ability to detect low amplicons [96]. Byrnes et al. reported a multiplexed and extraction-free amplification system that uses the CDC singleplex targets of coronavirus and can minimize the complexity and reagent usage, as well as the necessity for skilled technicians to operate, and holds the potential to minimize generating false-negative RT-PCR results. The system has a LoD of 2 copies/μL with 86% and 100% sensitivity and specificity, respectively [97]. Husain et al. reported adding two different steps in RT-PCR testing: firstly, the extraction of RNAs from multiple nasopharyngeal or oropharyngeal samples from the same person and the creation of an RNA pool to be used for RT-PCR testing; and secondly, the addition of nasal goblet cells, absorptive enterocytes, and type-II pneumocyte-specific control marker genes in RT-PCR testing to reduce false-negative results [98].

Laboratory design: The WHO Laboratory Biosafety Manual (LBM), which outlines the highest standards and establishes trends in biosafety, is widely used across all levels of clinical and public health laboratory settings and other biomedical sectors worldwide [99]. A molecular laboratory must be organized in such a way as to prevent the production of inaccurate results. There should be at least two places for PCR testing: pre-PCR and post-PCR areas. It is recommended that one room or space be utilized primarily for pre-PCR and that this area be partitioned into two further rooms or regions. One should be used exclusively for creating the PCR master mix, while the other should be used for sample preparation or extraction [100]. Consumables and tiny equipment should be available in the space, such as a mini-centrifuge, vortex, pipettes, tips, tubes, etc. There needs to be a minimum of two refrigerators used exclusively for their designated purposes, one for storing the kits and another for storing the samples [32]. The first room, used for pre-PCR activities, should be physically isolated from the second room, which will be used for post-amplification activities such as analysis. In this room, the thermal cyclers used for amplification will be operating, and any apparatus required for post-PCR analysis should be stored in the separate room designated for that purpose. Post-PCR preparation should also involve using smaller equipment, such as a mini-centrifuge, vortex, pipettes, tips, and tubes [100]. The area for master mix preparation, the same processing zone, the location for amplification or template addition, the room for detection, and the donning and doffing zone all should be singleplex isolated. The routes for the master mix room, extraction room, and detection area must be unidirectional or one-way directional [100]. A successful flow across geographically distinct places is crucial to guarantee the lowest possible contamination risk, including PCR amplicon carry-over contamination [4].

Heating, ventilation, and air conditioning (HVAC) system and maintenance of negative and positive air pressure: Contamination with infectious agents in the testing facility can be avoided by adequately applying heat and air passage and employing an HVAC system for air conditioning. However, infectious diseases like measles, tuberculosis (TB), chicken pox, influenza, smallpox, and severe acute respiratory syndrome (SARS) can spread through ventilation systems [101]. Patients or technicians may be more likely to get the illness if facilities are provided through shared ducts without using high-efficiency particulate air (HEPA) filters in a hospital or a diagnostic laboratory area [102]. During the molecular diagnosis of these samples, the laboratory air may also be contaminated. The contaminated air is a risk for laboratory personnel and may also be responsible for false results. HVAC systems are designed to guard against nosocomial infections, such as measles, an RNA virus-caused airborne disease, and keep the air clean. However, HEPA filters must be utilized in settings and outlet-exhausted tubes in such circumstances [103]. Positive pressure rooms retain an internal pressure comparatively higher than the outside.

In contrast, the unfavorable pressure rooms maintain a lower air pressure inside to facilitate airflow from outside. These protective room settings, known as 'airborne infection isolation rooms,' inhibit contaminants' transmission to secure the working zones' sterility. These room settings are crucial components of a wide range of medical and research facilities because they aid in maintaining clean conditions [104]. In a molecular laboratory that deals with infectious samples, the laboratory sample processing room should be set up with 100% exhaust negative air pressure, and the master mix room should be set up with a positive air pressure system [105]. Moreover, they shouldn't share the common ducts.

Equipment and trained personnel: Appropriate one-time-use clothing like personal protective equipment, gloves, masks, coats, mob caps, and goggles should be available in each room and often changed by the users [106]. Each location is responsible for ensuring they have the appropriate tools, disposable devices, and pipettes that do not contain aerosols or reagents [100]. Each work area must have its own distinct collection of equipment and apparatus, such as racks, test tubes, test tube stands, micropipettes, centrifuges, etc., to reduce the possibility of cross-contamination [106]. The work should be carried out within a biosafety level-two cabinet with the airflow directed downwards, pushing aerosols away from the top of the reaction tubes towards the base of the cabinet [107]. The detection procedure should be performed by trained personnel. The technologists must be alert to contamination during the working period. They need to know the potential for transferring amplification products from contaminated rooms to clean rooms via the wearer's hair, spectacles, jewelry, and clothing [100]. The working materials of the different areas should not be mixed to avoid RNA contamination.

Routine laboratory cleaning: It is imperative to consistently maintain cleanliness in work environments, ensuring that all workspaces, equipment, and commonly handled items such as doorknobs, telephones, and refrigerators are routinely sanitized [33]. Cleansers, including nitric acid (HNO3) and ethanol, poorly remove contaminated DNA and RNA. After applying 10% bleach to the exposed surfaces of the instruments, it is recommended to use isopropyl alcohol to eliminate any remaining traces of bleach thoroughly. The disposable cartridge should be disposed of according to the laboratory's standard methods [108]. Wipe tests should be regularly checked for RNA contamination because Cone et al. noted that the inhibitory compounds forming on lab surfaces can lead the wipe test to a false-negative result [109]. Laboratory personnel are advised to perform duplicate tests on each wipe-test sample utilizing a routinely amplification-positive control [110].

Ultra-violet light irradiation and autoclaving: It is also suggested that ultraviolet light eliminate any contaminated DNA or RNA. The UV light might shield surfaces like lab benches, floors, equipment, microcentrifuge tubes, and racks against DNA/RNA contamination [106]. The principle is based on the ability of UV radiation to cause covalent changes in DNA, such as the formation of thymidine dimers within DNA fragments. As a template for further amplification, this contaminated nucleic acid is inactive. For the intended function, a 254 and 300 nm wavelength combination is advised to continue for five to 20 minutes [111]. Several publications reported that this technique has a degree of success [112]. Another issue that needs to be considered is that UV irradiation's effectiveness is influenced by the distance between the DNA and the UV source [113]. However, some restrictions have been placed on UV irradiation. Oligonucleotide primers and enzymes like Taq polymerase can be damaged by UV radiation, inhibiting their activity [114]. Even though certain restrictions are associated with UV irradiation, it should be a fundamental component of every PCR lab. UV light should also be used at the entry and exit ducts of the HVAC system.

Disposals should be autoclaved before they are disposed of outside the laboratory. Research found that bacteriophages, adenoviruses, poliovirus, and echovirus are destroyed faster when planted in autoclaved water than in other water [115]. Some instruments may routinely be autoclaved for 30 min under 15 psi of pressure at 121 °C [115]. Autoclaving may reduce false results in molecular laboratories and prevent environmental contamination outside the laboratory [115].

Avoiding contamination and the process of decontamination: There are several potential sources of contamination risk, ranging from the receipt of clinical specimens through molecular analysis. These sources should be identified for each molecular test, and suitable control mechanisms should be used to reduce each risk [4]. There are two ways to prevent false-positive findings from nucleic acid amplification tests. One is that contamination hazards should be kept as low as possible, and another is that contamination should be eliminated as soon as possible if it occurs [116]. To reduce contamination, one must be careful with chemicals, disposables, laboratory equipment, and the surrounding environment [117]. To avoid the spread of aerosols, working tubes and bottles should be opened carefully [106]. Twenty-four hours' work break and proper cleaning of nasopharyngeal areas before sampling, adequate disinfection of the sampling site, and ensuring professional training are recommended to reduce the generation of false-negative results by inhaling aerosolized vaccines by healthcare personnel [25].

Proper management of waste materials and potential sources of contamination, such as disposable equipment, including pipette tips, buffer solutions, and experiment tubes, is essential to minimize the generation of aerosols. Plastic contaminated by nucleic acids must be gathered in disposable bags and sealed immediately after work completion [32]. All reagents that can cause deadly reactions should be disposed of, and all potential reagents should be replaced after determining which one is responsible for contamination. After that, thoroughly cleaning the laboratory with approved cleaners known to obliterate nucleic acids is needed [118]. If the contamination persists, personnel must do the clinical evaluation, and the procedure should be redesigned to include various genomic parts of the pathogen [118]. Some chemicals are well known for reducing contamination; however, some studies show the opposite. For instance, using 1 M HCl for decontamination may not work correctly [106]. Schmidt et al. discovered that even after 2.5 hours of treatment with HCl, the nucleic acid contamination was not destroyed [119]. Prince and Andrus have also described that even 2 M HCl did not yield the destruction of nucleic acid, which was detectable by PCR (100); however, this chemical may be used to reduce surface tension [106]. Using sodium hypochlorite to decontaminate the testing zone and the instruments in the molecular and RT-PCR laboratories where COVID-19 testing occurs is recommended. Applying 0.08% of sodium hypochlorite solution for five minutes was suggested by Prince et al. to degrade nucleic acid into tiny fragments, whereas another study reported incubating with an increased concentration of this chemical (0.4% w/v) for 30 minutes to obtain sufficient decontamination [120]. However, after incubation, the 200-base RNA target could no longer be detected using nucleic acid sequence-based amplification.

Nonetheless, a daily 0.4% (w/v) solution incubated for five minutes yielded significantly better outcomes [32]. Most studies suggest that 10% sodium hypochlorite solution (bleach) should be used for cleaning workstations where bleach can destroy nucleic acids by oxidative means and prevent its amplification following PCR reactions [121]. Furthermore, the contaminated tray must be taken to a clean area, where it can spend the night in a 2% to 10% bleach solution after thoroughly cleaning [100]. Another consideration that should be considered is that bleaching may cause harm to the plastic equipment and create a spot on the cabinet. So, after the bleach treatment, the equipment and workplace required cleaning.

Include both control in the procedure and the automated testing platform: Incorporating positive and negative control into the procedure is necessary to validate that the extraction and amplification steps are carried out appropriately. A control that does not include a sample is known as a negative control or control without a template, abbreviated as NTC. Incorporating a negative control provides insights into the potential presence of contamination in reagents, consumables, or the immediate environment [122]. Positive control indicates whether the kit is working correctly or not. Negative controls are critical in identifying potential contamination points within diagnostic tests, specifically in DNA extraction and PCR-related contamination cases.

Conversely, positive controls, particularly those utilized in DNA extraction procedures, are instrumental in detecting PCR reaction inhibition resultant from inhibitory chemicals remaining with DNA extracted from the test specimens, for instance, sodium polyanethole sulfonate co-elution in cases of blood specimens [123]. Automated testing platforms may be set up to reduce false results. This platform will increase diagnostic accuracy and help minimize the possibility of human mistakes in the testing performance [14].

Repeated testing: When patients have COVID-19 symptoms but get false-negative results, the sample must be re-tested. However, it needed to be clear under what conditions repeat RT-PCR testing would be required [124]. As per recent recommendations issued by the World Health Organization (WHO), it is advised to request repeat testing for patients exhibiting persistent symptoms of COVID-19, including collecting a swab from the lower respiratory tract [125]. Repeating the test with new nasopharyngeal samples is advisable in cases of clinical solid suspicion [64]. After consecutive false-negative RT-PCR test results among patients' post-clinical recovery from COVID-19 in Wuhan, China, Wang et al. suggested getting three successive negative results before discharging patients from hospitals [126]. However, correlation with the patient's clinical information is necessary; long-term COVID-positive patients can be confused with an active infection; during the beginning of the pandemic, some patients were confined to hospitals for prolonged and unnecessary times after their recovery because they continued to be presenting positive tests for residual RNA, increasing the saturation of health systems. In addition, it is advised to employ the fewest number of cycles possible when carrying out the amplification reactions. To create the amplicon required to get the desired results, any changes to the technique, especially those made to tests used for diagnostic reasons, must be validated [32].

Future recommendations for these issues

This review relied on the data regarding the issues contributing to erroneous results in the RT-PCR-based diagnosis approach, which was extensively applied during the COVID-19 pandemic. Although RT-PCR is a powerful diagnostic tool, it is prone to several limitations, e.g., the strict requirement of specialized equipment and expertise regarding the operation of the apparatus and knowledge regarding the reasons and mitigation strategies of false-positive and false-negative results. The severity of the pandemic, the enormous magnitude of the infected patients, and the necessity of rapid diagnosis created an unprecedented challenge and pressure on the RT-PCR technique, laboratory facilities, and personnel, which might eventually fail in strict adherence to the RT-PCR protocol and thus the generation of false results. Repetition of the error-prone steps and further investigational approaches must be performed to correctly identify the points leading to erroneous results. Adequate measures must be taken to avoid potential contamination, technical errors, and false-positive results.

In contrast, precautions and strict adherence to the standard protocols for specimen collection, transportation, storage, and handling are necessary to minimize false-negative results. Strict maintenance of the WHO Laboratory Biosafety Manual (LBM) and other WHO and CDC guidelines related to the RT-PCR standard protocol and minimization of its false results is highly recommended. Performing additional tests, e.g., CT scans and hematological tests, variations in conventional RT-PCR platforms, e.g., RT-nested PCR, digital droplet PCR, including further steps in RT-PCR testing or the development of rapid point-of-care tests, can be applied to improve the accuracy of RT-PCR along with mitigating false results. Besides, updated serological assays can be applied parallelly with the standard RT-PCR technique to resolve this problem. Adequate laboratory facilities and monitoring systems, trained personnel, routine checking and calibration of the RT-PCR equipment, and rapid and properly designed transportation and storage systems for specimens must be implemented to minimize the risks of generating false results. Moreover, more trials must be performed to create an efficient, less error-prone, standard RT-PCR protocol to meet the complications of general situations and emergencies like the COVID-19 pandemic.

Limitations of the study

This review was mainly based on discrete information generated during the emergency of the COVID-19 pandemic, which might have resulted in a lack of generalization. Since this study was designed for a narrative review, systematic search approaches were not applied, which might have led to the exclusion of information that might have seemed relevant to this study. The lack of statistical analysis limited the review's findings, resulting in a lack of quantitative data on false-positive and false-negative RT-PCR results.

## Conclusions

Molecular diagnostic tests like RT-PCR are vital for COVID-19 detection but can produce misleading, false outcomes resulting from contamination, handling errors, or viral evolution. This review discusses factors contributing to inaccurate molecular test results during the pandemic. It proposes solutions to improve reliability, like implementing stringent quality control and contamination prevention protocols, including proper cleaning, workflows, reagents, dedicated equipment, and frequent process monitoring. Upgrading primer designs regularly to match viral mutations also enhances the detection of emerging variants. A multifaceted testing approach using clinical, epidemiological, and molecular data provides added perspective. Accurate, reliable molecular testing is critical for proper COVID-19 diagnosis and management. Implementing strategies aimed at fortifying quality control, sample handling, laboratory protocols, and primer designs makes enhancing the efficacy and accuracy of RT-PCR and other significant molecular diagnostic assays possible.
