# Correction: Strategies to Overcome Erroneous Outcomes in Reverse Transcription-Polymerase Chain Reaction (RT-PCR) Testing: Insights From the COVID-19 Pandemic

**DOI:** 10.7759/cureus.c199

**Published:** 2024-11-21

**Authors:** SM Shafiul Alam Sajal, Dewan Zubaer Islam, Shahad Saif Khandker, Elizabeth Solórzano-Ortiz, Manal Fardoun, Md Firoz Ahmed, Mohd. Raeed Jamiruddin, Nafisa Azmuda, Miral Mehta, Santosh Kumar, Mainul Haque, Nihad Adnan

**Affiliations:** 1 Department of Biochemistry and Molecular Biology, Jahangirnagar University, Dhaka, BGD; 2 Department of Microbiology, Jahangirnagar University, Dhaka, BGD; 3 Department of Microbiology, Gonoshasthaya Samaj Vittik Medical College, Dhaka, BGD; 4 Department of Chemical, Biological, Biomedical and Biophysical Research, Mariano Gálvez University, Guatemala City, GTM; 5 Department of Pharmacology and Toxicology, Faculty of Medicine, American University of Beirut, Beirut, LBN; 6 Department of Pharmacy, Bangladesh Rural Advancement Committee (BRAC) University, Dhaka, BGD; 7 Department of Pedodontics and Preventive Dentistry, Karnavati School of Dentistry, Karnavati University, Gandhinagar, IND; 8 Department of Periodontology and Implantology, Karnavati School of Dentistry, Karnavati University, Gandhinagar, IND; 9 Department of Pharmacology and Therapeutics, National Defence University of Malaysia, Kuala Lumpur, MYS

This article has been corrected as follows to replace reference 69 with the correct information.

Original reference: Do COVID-19 Tests Still Work After They Expire? Here's How to Tell. (2023). Accessed: October 12, 2024: https://www.cbsnews.com/news/covid-test-expiration-dates-fda-extension-accurate.

New reference: Facente SN, Dowling T, Vittinghoff E, Sykes DL, Colfax GN: False positive rate of rapid oral fluid HIV tests increases as kits near expiration date. PLoS One. 2009, 4(12):e8217. 10.1371/journal.pone.0008217.

